# Construction and in vitro evaluation of pH-sensitive nanoparticles to reverse drug resistance of breast cancer stem cells

**DOI:** 10.1007/s12672-024-00873-w

**Published:** 2024-01-29

**Authors:** Weinan Li, Yuhan Fu, Jialin Sun, Hexin Gong, Ru Yan, Yanhong Wang

**Affiliations:** 1 School of Pharmacy, Heilongjiang University of Chinese Medicine, Harbin, China; 2 Key Laboratory of Basic and Application Research of Beiyao, Heilongjiang University of Chinese Medicine), Ministry of Education, Harbin, China; 3 Postdoctoral Research Station, Heilongjiang University of Chinese Medicine, Harbin, China; 4 Biological Science and Technology Department, Heilongjiang Vocational College for Nationalities, Harbin, China

**Keywords:** Breast cancer stem cells, pH-sensitive, Multidrug resistance, Nanoparticles, P-glycoprotein

## Abstract

Breast cancer is a major threat to safety and health of women. The breast cancer stem cells (BCSCs) have multi-drug resistance to chemotherapy drugs, which leads to chemotherapy failure. We proposed a strategy of delivery of tumor-killing drugs and a resistance reversal agent, to enhance inhibition of BCSCs. Here, schisandrin B (SchB)/AP NPs are constructed using acid-grafted-poly (β-amino ester) (ATRA-g-PBAE, AP) grafted polymer nanoparticle encapsulated SchB, with pH-sensitive release function. This drug delivery system has good pharmacological properties and can increase the SchB release with the decrease of pH. The NPs showed cytotoxic effects in reversing ATRA resistance to BCSCs. Lysosomal escape was achieved when the nanoparticles were taken up by BCSCs. In addition, we found that NPs may reverse MDR by inhibiting the expression of P-glycoprotein (P-gp) and affecting the energy supply of drug efflux. This study provides a nanodelivery therapy strategy that reverses BCSCs multidrug resistance (MDR) and demonstrates that it did so by interfering with cancer cell energy metabolism. Therefore, the co-delivery strategy of ATRA and SchB provides a new option for the treatment of breast cancer.

## Introduction

Breast cancer is the most common malignant tumor in women and has become a serious public health problem in the world today. It has been reported that cancer stem cells are the pivotal cause of malignant tumors [1, 2]. BCSCs are the origin of breast cancer, causing tumors to grow or form new tumor focus. BCSCs have more obvious tumorigenic ability than normal breast cancer cells [3]. Therefore, targeting BCSCs has become a critical approach in the fight against breast cancer.

Peptidyl-prodyl cis-trans isomerase 1 (Pin1) is a tumor-specific enzyme [4]. The overexpression of Pin1 in tumor cells was positively correlated with tumor stem cells progression [5]. Clinical studies have found that the expression level of Pinl can affect the occurrence and prognosis of breast cancer. Pin1 is highly expressed in tumor cells, and the expression level in BCSCs is 30 times higher than in common tumor cells [6, 7]. ATRA is a natural derivative of vitamin A. Wei et al. proved that ATRA could kill tumor cells by inhibiting Pin1 through high-throughput screening experiments [8]. Our previous study found that ATRA showed good toxicity in breast cancer cells, but its toxicity to cancer stem cells was too low [9]. Thus, the inhibition of Pin1 alone is not effective against BCSCs.

BCSCs have strong drug resistance due to the “drug pump” effect of high expression of drug-resistant proteins on their surface. Under the action of drug-resistant proteins, drugs entering cells are expelled from cells, and drug resistance occurs [10, 11]. Importantly, BCSCs were often more resistant to drugs than ordinary tumor cells. ATRA showed lower toxicity in tumor stem cells compared to normal tumor cells, which may be an obstacle to drug resistance [12]. For enhancing the effect of ATRA on breast cancer stem cells (Michigan Cancer Foundation-7 (MCF-7)-induced MS cells were used in this study), it is necessary to load MDR reversal agent to reverse resistance.

Currently, the multidrug resistance mechanism of membrane transporters is critical [13]. ABC transporter-mediated MDR of tumor cells mainly uses the hydrolysis of ATP to obtain energy to expel drugs outside the cells. P-gp is the main component of ABC membrane transporter [14]. P-gp uses the hydrolysis of ATP to obtain energy, and expels drugs entering the cell outside the cell [15]. Many natural products can overcome multidrug resistance, such as quercetin and SchB [16]. SchB has been reported to restore drug sensitivity to MCF-7/MDR1 cell lines containing stable P-gp expression [17]. SchB may increase cytoplasmic drug content by inhibiting P-gp function. SchB has a similar chemical structure to P-gp inhibitors [18]. Notably, in addition to tumor cells, P-gp is expressed in other normal tissues. Therefore, introducing nanotechnology to deliver insoluble SchB to BCSCs efficiently is even more necessary.

Nanoparticles are widely used in cancer treatment as delivery systems to deliver drugs to their destination. In cancer treatment, nanoparticles can be designed as drug delivery systems. Based on the EPR effect, nanocarriers deliver anti-cancer drugs precisely to the tumor site, thereby reducing damage to healthy tissue. Moreover, nanoparticles can also be used to improve the solubility and stability of drugs, prolong the half-life of drugs, and thus enhance the therapeutic effect. Importantly, nanoparticles can combine a variety of therapeutic agents in one, to achieve multi-drug synergies. For example, PBEA is a class of pH-sensitive polymer carrier materials [19]. It can be protonated under acidic conditions, resulting in structural changes, resulting in the release of internal drugs. This characteristic is just suitable for the slightly acidic environment of the tumor cell lysosomes (pH 5.5). Although many literatures have realized resistance reversal, few literatures have designed tumor suppressor drug carrier and reversal agent in the same nanodelivery system and realized energy metabolism intervention, especially targeted delivery of multi-drug reversal agent SchB.

**Scheme 1:**
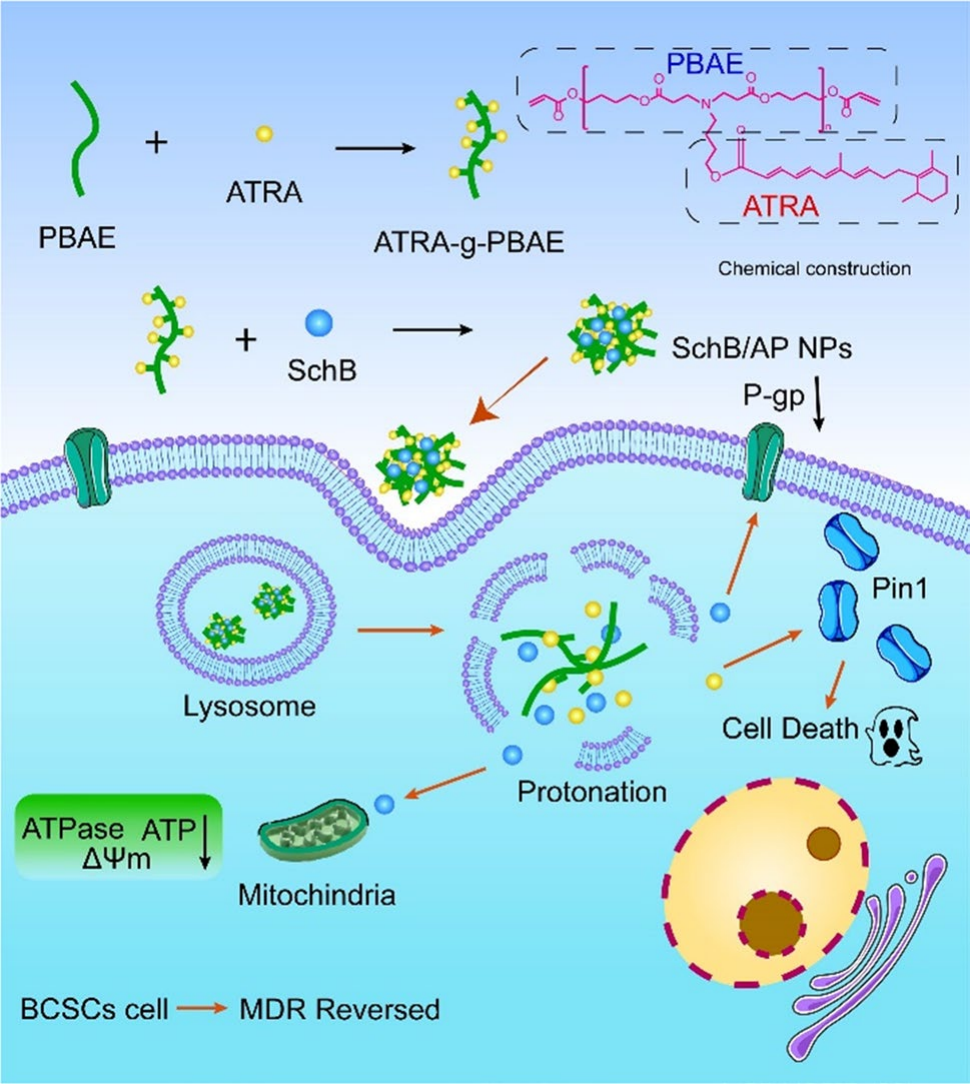
Construction of SchB/AP NPs and the mechanism for reversing MDR against BCSCs. The SchB/AP NPs can co-deliver ATRA and SchB into the cells, enabling SchB lysosome escape and responsive release by the pH-sensitive function of the carrier. Importantly, NPs can inhibit the expression of P-gp and weaken the energy metabolism function of cells, ultimately achieving the reversal of MDR.

Herein, to reverse BCSCs resistance and enhance the anticancer effect of ATRA, a pH-sensitive polymer loaded with a hydrophobic reversing MDR agent SchB was constructed (Scheme 1). Particularly, the ATRA-g-PBAE was used as a carrier to encapsulate the active molecule SchB to obtain SchB/AP NPs. Passive target delivery of SchB reverses MDR by inhibiting ATPase activity and mitochondrial function affecting P-gp energy supply and transport, which was the novelty of the NPs. The delivery of SchB to reverse BCSCs resistance to ATRA provided a new option for breast cancer treatment. This indicates that SchB/AP NPs have strong application potential in synergistic enhancement of BCSCs inhibition.

## Materials

3-(4,5)-dimethylthiahiazo (-z-y1)-3,5-di- phenytetrazoliumromide (MTT) was from APExBIO Co., Ltd. (Houston, USA). PBS was from BioChannel Biological Technology  Co., Ltd. (Guangzhou, China). Cyanine 5.5(Cy5.5) and Coumarin-6 (C6) were from Aladdin Co., Ltd. (Shanghai, China). Lyso-Tracker Green, bicinchoninic acid (BCA), ATPase activity assay kit were from sigma Co., Ltd. (Shanghai, China). Mitochondrial membrane potential JC-1 assay kit was from Beijing Solarbio Science & Technology Co., Ltd. ATP content activity assay kit was from Grace Biotechnology Co., Ltd (Suzhou, China). FITC-labeled sheep anti-rabbit IgG antibody was from GeneTex Co., Ltd (Beijing, China). P-glycoprotein rabbit antibody was from Zen bioscence (Chengdu, China). Dulbecco’s modified Eagle’s medium F12 (DMEM-F12) was from BDBIO Co., Ltd. (Hangzhou, China). The cell culture plates were from NEST Biotechnology Co., Ltd. (Wuxi, China). The microwell plates were from CellPro Biotechnology Co., Ltd. (Suzhou, China). HAKATA Fetal bovine serum was from Chuanqiu Co., Ltd. (Shanghai China). Human Insulin protein was from ABclonal Technology Co., Ltd. (WuHan, China). The cells culture bottles were from SAINING (Suzhou, China). Cryopreservation was from Sevenbio (Beijing, China). Recombinant human FGFb was from Novoprotein Co., Ltd. (Shanghai, China).

MCF-7 cells (for MS cells formation) were obtained from the BNCC Co., Ltd. (Beijing, China). Serum-free MS culture medium was prepared by the following: The precise weight of 200 mg bovine serum protein was dissolved in 50 mL DMEM-F12 medium. Then 0.1 mL EGF solution (10 µg/mL), 0.1 mL bFGF solution (10 µg/mL), 0.5 mL antibiotics solution, and 0.5 mL insulin were added. After 0.22 μm filtration membrane, a serum-free medium was obtained.

MCF-7 stem cells (MS cells) were formed in the following way: take MCF-7 cells that have reached the logarithmic growth phase, wash them twice with PBS, and then digest the cells with 2 mL of trypsin. Centrifuge the cells twice, each time throwing away the supernatant before adding serum-free medium. Finally, resuspend the cells in a low-adsorption plate with 4 mL of serum-free MS culture medium. Cells spheroidized at 37 ℃,5% CO_2_ were observed under a microscope and passaged every 5 days. In our previous experiments, the phenotype of MS cells has been verified by flow cytometry, and the formation of MS cells has been confirmed [18].

## Method

### The preparation of SchB/AP NPs

ATRA-g-PBAE prodrug polymer was obtained from previous experiments by our research group [9]. PBAE and ATRA-g-PBAE were respectively synthesized by michael and esterification reaction. The EDC·HCl and DMAP were added to the ATRA solution. Then, the PBAE solution was dropped into the mixture and reacted for 48 h. The purification process involved is the use of cold ether to precipitate excess ATRA. Then, to remove the EDC·HCl and DMAP, the fluid was transferred to a dialysis bag for dialysis for 24 h. The final product ATRA-g-PBAE was obtained by vacuum drying. Cy5.5-PBAE was prepared by the same method, only by replacing ATRA with Cy5.5.

SchB/AP NPs were prepared from the solvent volatilization method [20]. Briefly, a certain mass of ATRA-g-PBAE prodrug polymer carrier and SchB (20% carrier mass) were precisely weighed. The organic phase was mixtured by dissolving the carrier and SchB in acetone. According to the experimental design scheme, the organic phases with different carrier concentrations (mg/mL) and the feeding drug (SchB) ratio (%) were prepared. The above organic solution was slowly dripped into 15 mL of 0.5% poloxamer 188 aqueous phase. Then the solution continued stirring at 25 ℃ until the organic solvent evaporated. After filtering through a 0.22 μm filter membrane, the SchB/AP NPs solution with blue opalescent light was obtained. The preparation of C6/AP NPs is the same as the above, only C6 replaced SchB.

### Experimental design of the preparation of the SchB/AP NPs

The factor and level of Central Composite Design Response Surface Model (CCD RSM) were determined according to the preliminary experiment. ATRA-g-PBAE carrier concentration (mg/mL) and the feeding drug (SchB) ratio (%) were set as A and B, respectively. Particle size (Y_1_) and encapsulation rate (EE) (Y_2_) were used as response parameters. The NPs were prepared according to the preparation method in Sect. 3.1 for other conditions. Meanwhile, the response surface figure was drawn with the results data, and the optimal scheme was predicted. The experimental design scheme is shown in Table 1.

**Table 1:** Design scheme of CCD RSM

Level	Factor	
	A (mg/mL)	B (%)
-α	5	10
-1	10	15
0	15	20
1	20	25
α	25	30

### The encapsulation efficiency and loading capacity

1 mL of the prepared SchB/AP NPs solution was centrifuged at speed (10,000 rpm) for 60 min. The obtained supernatant was absorbed and methanol was added for demulsification. The solution was further demulsified by an ultrasonic cell crusher. Afterward, the demulsified solution was placed in a 5 mL volumetric bottle and filled with methanol to the scale. The liquid obtained by filtration through a 0.22 μm filter determined the SchB content as W_a_. In addition, 1 mL prepared SchB/AP NPs solution was taken for all above operations except centrifugation, and SchB content was determined, denoted as W_b_. The centrifugal precipitate above was taken and then its total mass was weighed after vacuum drying, denoted as W. All SchB contents were determined by high performance liquid chromatography (HPLC) (Shimadzu, 3100, Japan). The EE% and the drug loading capacity (LC)% were computed by the following formula:

$$\text{E}\text{E}\text{\%}=\frac{{\text{W}}_{\text{b}}-{\text{W}}_{\text{a}}}{{\text{W}}_{\text{b}}}\times 100\text{\%}$$

$$\text{L}\text{C}\text{\%}=\frac{{\text{W}}_{\text{b}}-{\text{W}}_{\text{a}}}{\text{W}}\times 100\text{\%}$$

### Characterization of the SchB/AP NPs

In order to obtain the appearance of the SchB/AP NPs, the transmission electron microscope (TEM) (JEOL JEM, 4378, Japan) was used to observe the morphology. The specific operation method was as follows: a little SchB/AP NPs solution prepared above was added to the 200-mesh copper net by drops. After 3 min, the excess liquid was absorbed by filter paper. After drying at room temperature, the morphology of the NPs was characterised by TEM.

The particle size and size distribution of SchB/AP NPs were determined by the Marvin particle size analyzer (Marvin, 2900, US). One drop of the NPs solution was added to the size cell. Finally, the average size and particle size distribution were recorded. The same method was used to measure the zeta potential.

### In Vitro SchB release

The optimal preparation of SchB/AP NPs was put into the given dialysis bags (Mw = 3,500). Moreover, the free SchB acted as the control group. Dialysis bags were transferred to 50 mL PBS solution containing 0.5% (m/w) polysorbate 80 at pH 5.5, 6.8, and 7.4. Water bath oscillations at 37 ℃ were used, samples were taken at different times. The sample solution was determined by the concentration of SchB by HPLC. The cumulative release was depicted and the cumulative release curve was plotted in accordance with following formula:

$$\text{Er}=\frac{{\text{V}}_{\text{e}}\sum _{\text{i}-1}^{\text{n}-1}{\text{C}}_{\text{i}}+{\text{V}}_{0}{\text{C}}_{\text{n}}}{{\text{m}}_{\text{d}\text{r}\text{u}\text{g}}}\times 100\text{\%}$$

where, E_r_, V_e_, C_i_, V_0_, C_n_ and m_drug_, were the cumulative release of SchB, the displaced volume of PBS, the concentration at the ith sampling (µg/mL), release medium volume (mL), the concentration at the nth sampling (µg/mL) and SchB content in drug-loaded NPs (mg), respectively.

### Serum stability of the SchB/AP NPs

The SchB/AP NPs were placed in serum contained DMEM at 37 ℃ to investigate the stability of the NPs. The mixture was shaken in a shaker, and 1 mL of the sample was aspirated at 0, 6, 12, 36, 24 and 48 h for particle size determination. The particle size was measured according to the particle size determination method in Sect. 3.4.

### The parameter of HPLC

The SchB was measured by HPLC fitted with a Prominence LC-20AD at 220 nm. The experimental result was detected on a Diamonsil C18 column (4.6 mm × 250 mm, 5 μm). The mobile phase A and B were respectively 15% phosphoric acid aqueous solution and 85% methanol. Moreover, the flow rate was 0.8 mL /min. Measured method of SchB concentration section “3.3 and 3.5” was according to those conditions above.

### In vitro cytotoxicity

In vitro cytotoxicity was detected by MTT. MS cells were seeded in 96 wells at a density of 7 × 10^3^ cells per well and incubated at 37 ℃ for 24 h at 5% CO_2_ in an incubator. The free solution of the drug was a blank control group. The pharmaceutical solution was taken into certain wells in the experimental group. Cell culture grade 0.05% DMSO was added to the wells to aid dissolution. Each concentration was made three parallel while leaving blank wells. After that, the cell culture plates were placed in the incubator for 48 h. Finally, the absorbance value was detected at 490 nm with a multifunctional microplate reader (JD-SY96A, JingDao, China). The IC_50_ at which the reversal agent SchB was added to certain wells was denoted as IC_50_ (*A/S*). The Reversal Factor (RF) was calculated according to the following formula:

$$\text{R}\text{F}=\frac{{\text{I}\text{C}}_{50\left(\text{F}\text{r}\text{e}\text{e} \text{A}\text{T}\text{R}\text{A}\right)}}{{\text{I}\text{C}}_{50(\text{A}/\text{S})}}$$

### Cellular uptake of the SchB/AP NPs

To verify the cellular uptake performance of the carriers of the NPs, Cy5.5-g-PBAE NPs were used to replace ATRA-g-PBAE NPs. Besides, to testify the cellular uptake ability of SchB /AP NPs, C6/AP NPs were used instead SchB /AP NPs. The NPs preparation method was referred to the Part 3.1. MS cells were digested and seeded in 6-well cell culture plates at a density of 2.5 × 10^4^. C6/AP and Cy5.5-g-PBAE NPs were diluted in the serum-free medium that were added to the wells and incubated for 0.5, 2 and 4 h, respectively. Pre-chilled PBS was added at the end of the incubation to stop cell uptake. PBS washed the cells twice to remove drug interference and then placed them under a fluorescence microscope (Laika, 5699, Germany) to observe cell uptake. Finally, flow cytometry (Beckman, CytoFLEX, China) was used to determine the intensity of cells fluorescence at different times.

### Lysosome co-localization

Coverslips were placed in 6-well plates, and MS cells were seeded on them at 10^5^ cells/well. After overnight culture, 2 mL Cy5.5-g-PBAE NPs solution was added into each well and incubated for 0.5, 1 and 2 h. Intracellular lysosomes were labeled with 200 µL Lyso-Tracker Green lysosome fluorescent probe. After 30 min of incubation, cells were fixed with 4% paraformaldehyde, and placed under confocal laser scanning microscope (CLSM) (Olympus, FV3000, Germany) to observe the intracellular drug delivery of MS cells.

### The mechanism of MS cells reversing MDR

#### Determination of P-gp expression

MS cells with strong growth were incubated for 24 h. After 24 h incubation in the incubator, 2 ml PBS was washed twice. The cells incubated without drug solution were set as the control group, and the cells incubated with different preparation groups were set as the experimental group. After 12 h incubation, centrifuge to collect the cells and wash twice with cold PBS. And the rabbit anti-human P-gp primary antibody was added and incubated at 4 ℃ protected from light. Then the mixture was centrifuged to remove unbound antibodies. Then, the already diluted FITC-labeled sheep anti-rabbit IgG secondary antibody was added to the centrifuge tube for staining. After 30 min of incubation at 4 ℃ in the dark, the mixture was centrifuged and discarded the supernatant. The excess fluorescent substance was removed by PBS cleaning, and the expression of P-gp was detected by fluorescent microplate reader. The excitation wavelength is 488 nm and the maximum emission wavelength is 525 nm. It is detected by the FL1 channel of flow cytometry.

#### Assay of intracellular protein content

The Bicinchoninic acid (BCA) method was to detect the protein concentration of MS cells in preparation for the ATPase activity assay and ATP content. Single-cell suspensions of logarithmically grown MS cells were collected and seeded in cell culture plates. After 24 h of incubation, the cells were rinsed twice with normal saline to prevent contamination of the sample with phosphorus. The solution of three different preparation groups was added into different holes respectively, and the blank medium without drugs was the control group. After 2 h of incubation, MS cells were resuspended in 0.3 mL saline. The cells were disrupted using a cell disruptor and diluted 3-fold with normal saline for assay. The protein concentration of samples was calculated by constructing the protein standard curve.

#### Measurement of ATPase activity

MS cells of logarithmic growth stage were collected and washed with normal saline 3 times. A drug-free medium was added into the culture hole as the control group. Three different preparation solutions were added to the other groups. The value of Na^+^-K^+^-ATPase, Ca^2+^-Mg^2+^-ATPase and T-ATPase were measured respectively. After incubation for 24 h, the supernatant was taken for ATPase activity determination. The absorbance value of 636 nm was measured. To calculate the activity of Na^+^-K^+^-ATPase, Ca^2+^-Mg^2+^-ATPase and T-ATPase, the ATPase content was calculated according to the following formula.

$$\text{ATPase activity} (\text{U}/\text{m}\text{g}\text{p}\text{r}\text{o}\text{t})=\frac{{\text{A}}_{1}-{\text{A}}_{0}}{\left({\text{A}}_{2}-{\text{A}}_{3}\right){\text{C}}_{\text{0}}}\times 6\times 7.8$$

Where, the A_1_, A_2,_ A_3_, A_0_ and C_0_, were determination group absorbance, standard group absorbance, blank group absorbance, control group absorbance and protein concentration of sample to be tested.

#### Assessment of mitochondrial membrane potential (ΔΨm)

MS cells were inoculated into 6-well plates with 5 × 10^4^ cells per well and cultured for 24 h. Then, the solution of three formulations was added. In addition, drug - free medium was added into the culture hole as the control group. After incubation for 2 h, the cells were re-suspended in 500 µL of 10 µg/mL JC-1 working solution and incubated at 37 ℃ for 20 min. Finally, the cells were washed twice with JC-1 staining solution and suspended in 500 µL buffer solution. The values of red fluorescence (λex = 525 nm / λem = 590 nm) and green fluorescence (λex = 490 nm / λem = 530 nm) were observed and determined by fluorescence microplate.

#### Assay of ATP content

ATP content was determined by ATP colorimetric assay kit. MS cells were cultivated with medium containing blank group and preparation group solutions, then incubated for 2 h. Subsequently, the ATP working solution was added and measured the absorbance value. In the other part of the sample, the protein content of the cells was through BCA method, and the ATP concentration was converted into the form of nmoL/mg protein to eliminate the error caused by the different number of cells per well. The absorbance of the treated samples was measured by the microplate reader. ATP_1_ was the measured data of the experimental group, and ATP_2_ was the measured data of the control group. The relative ATP activity is calculated as follows:

$$\text{Relative ATP activity}=\frac{{\text{A}\text{T}\text{P}}_{1}}{{\text{A}\text{T}\text{P}}_{2}}\times 100\%$$

### Statistical analyses

The experiment was repeated 3 times and the results are reported as mean ± SD (standard deviation). The experimental design and data processing of CCD-RSM was completed by Design Export 11. The analysis of experimental data was made using GraphPad Prism 11. The Student’ s t-test between 2 groups or more was with ANOVA test when more than 3 groups. Significance at *p < 0.05 or **p < 0.01 was acceptable.

## Results and discussion

### Optimization result of the SchB/AP NPs

RSM has been widely used in process optimization schemes, including the preparation of nanoparticles. As a means of experimental design, CCD is often used in conjunction with RSM (CCD RSM) to optimize the formulation of NPs preparation. CCD RSM was used to optimize the preparation of SchB /AP NPs. The experimental data were fitted with multiple linear and second-degree polynomial regression, and the results were as follows:

$$Y_{1} = 172.51 - 2.46{\text{A}} - 1.60{\text{B}} + 0.0075{\text{AB}} + 0.087A^{2} + 0.032B^{2} \,({\text{p < 0}}{\text{.05}})$$

$${Y}_{2}=45.90+1.57\text{A}+2.30\text{B}+0.0075\text{A}\text{B}-0.063{A}^{2}-0.061{B}^{2} \,({\text{p < 0}}{\text{.05}})$$

**Fig. 1:**
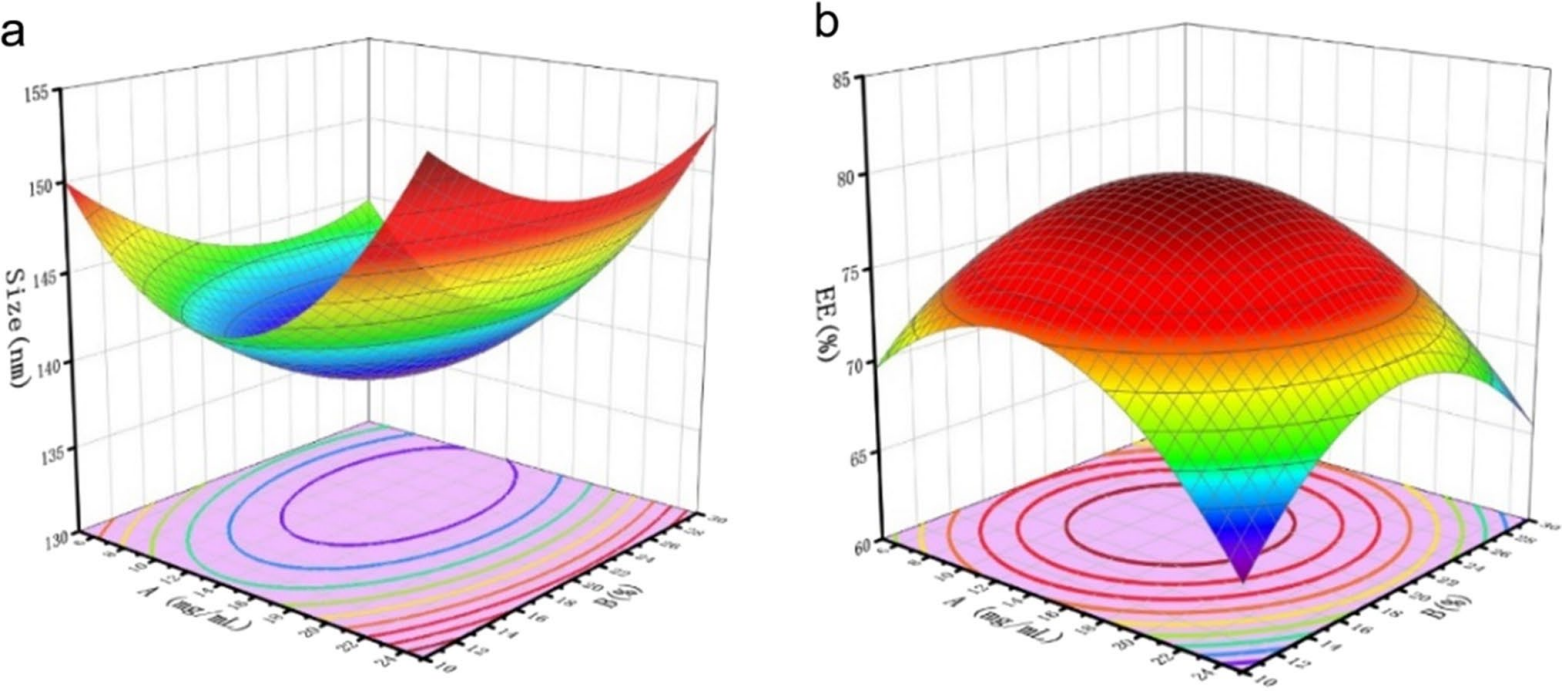
The response surface of Y_1_ (Size) (**a**) and Y_2_ (EE) (**b**) results. A is the carrier concentration (mg/mL). B is the feeding drug ratio (%)

The response surface in Fig. 1 was obtained by fitting the equation. Figure 1a is the change in particle size caused by factors A (carrier concentration) and B (feeding drug ratio), and Fig. 1b is the change of EE caused by factors A and B. It can be seen Fig. 1a that particle size gradually decreases and then increases with the increase of A and B. As shown in Fig. 1b, EE increased first and then decreased with the rise in values of A and B. The reason is that after the concentration of the carrier was too high, the organic phase was opposed to dispersing evenly in the water phase, and agglomeration occurred [21]. As the feeding drug ratio was too high, the carriers could not carry more SchB, resulting in the decline of EE. Thus, carrier concentration and feeding drug ratio should be maintained at a medium level to obtain lower particle size and higher EE. Finally, according to the software prediction, the optimal conditions are as follows: carrier concentration was 22.78 mg/mL, and feeding drug ratio was 19.32%. The optimal particle size is 146.5 nm and the EE is 73.89%. The best prescription verification results of SchB/AP NPs prepared by the optimal scheme were as follows: the particle size was 135.1 ± 0.96 nm, the LC was 5.01 ± 0.15%, the EE was 80.68 ± 1.15%. The actual result is similar to the size and EE predicted by the software.

Typically, the size of nanoparticles is designed to be around 100–200 nm. This is due to the presence of the enhanced permeability and retention effect (EPR), which means that the capillary epithelial cells near the tumor cells are not tightly arranged, resulting in pore sizes between 100 and 200 nm, while the capillary vessels in normal tissues are extremely tightly arranged [22]. Therefore, the nanoparticles will be selectively enriched in the tumor area.

### Characterization of the SchB /AP NPs

**Fig. 2:**
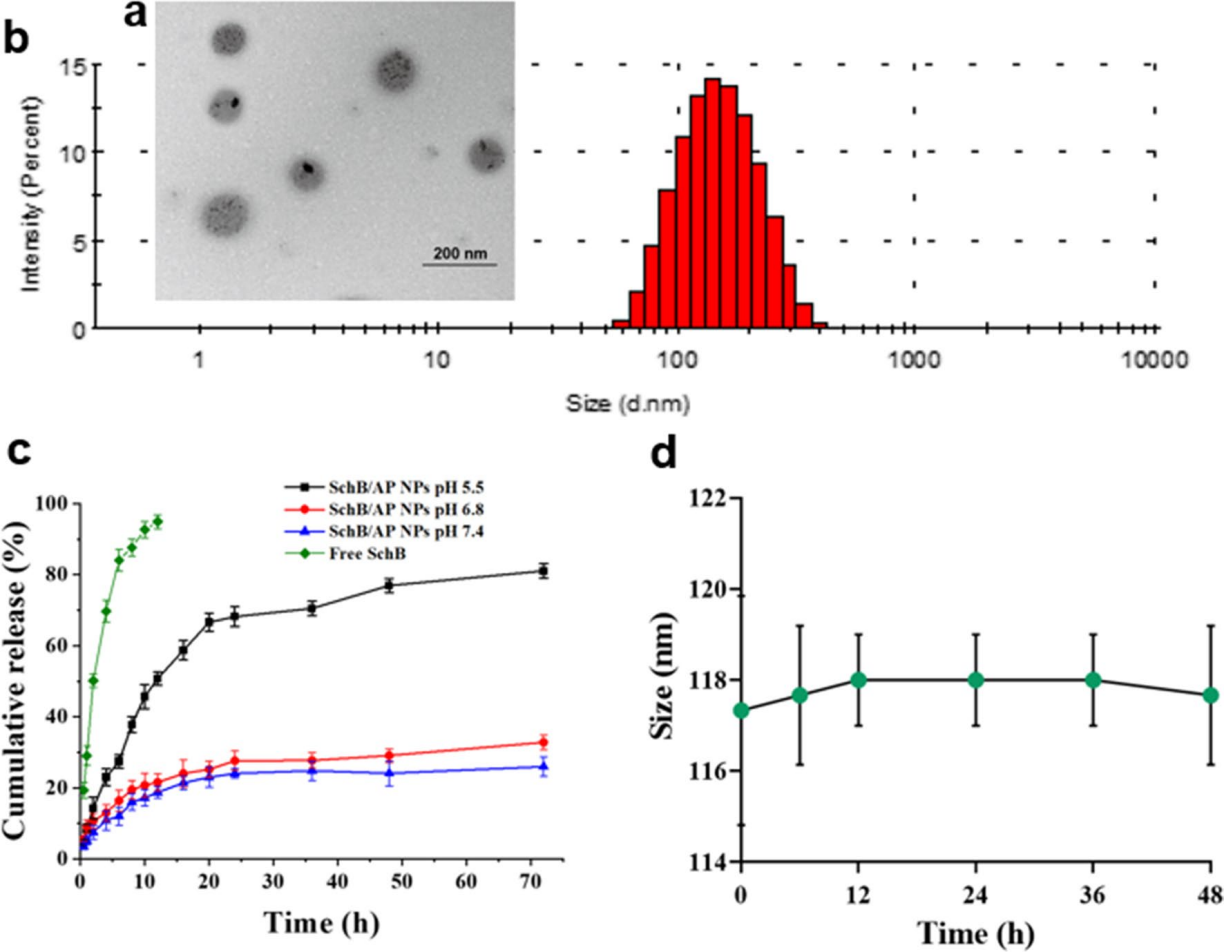
**a** The TEM figure and **b** size distribution (the scale bar was 200 nm); **c** The in vitro release courve of SchB and **d** serum stability of the SchB/AP NPs.

As shown in Fig. 2a, the TEM of SchB/AP NPs could be seen from the figure that the NPs were spherical-likeand monodisperse. The result showed that the hydrophobic ATRA-g-PBAE could condense into spherical form under the influence of hydrophobic action, and the stronger the hydrophobic action was, the smaller the particle size was. Monodisperse nanoparticles indicate that there is no aggregation between particles, which is extremely important for the distribution of nanoparticles in vivo [23]. Figure 2b shows the particle size distribution, which showed a particle size of 134.97 ± 0.25 nm. The results of the two characterization methods are consistent and similar to the size obtained by the optimal scheme. Thus, the SchB/AP NPs prepared by the optimal scheme have good pharmaceutical properties.

Sufficiently small particle size is beneficial to NPs to be passively targeted to the tumor and enriched within the tumor under the EPR effect [24]. The EPR effect relies on the high vascular permeability and low lymphatic outflow of solid tumors to achieve passive targeting and long-term retention of nanoparticles at the tumor site.

### In Vitro SchB release

To realize the study of drug release by NPs in tumor cells, pH 7.4, 6.8 and 5.5 were selected to simulate the pH of normal physiology in vivo, tumor microenvironment and the lysosomes, respectively [13, 25]. The release curves of SchB/AP NPs in different pH release media were shown in Fig. 2c. Free SchB was rapidly released from the dialysis bag to the medium solution within 12 h, indicating that the dialysis bag had minimal effect on the release of SchB. Within 48 h, the release of SchB reached 80% at pH 5.5. At pH 6.8, SchB release was finally 30%.

The pH of the lysosomes of tumor cells is 5.5, and experimental results show that the drug is rapidly released at this acidity. PBAE is a pH-sensitive polymer, which can undergo strong protonation under acidic lysosomal conditions [26]. At the same time, the protonation causes abundant chloride ions and water molecules to flow into the lysosome, resulting in osmotic pressure increasing and e lysosome rupturing [27]. Accordingly, after the nanoparticles are protonated in the lysosome, the released drugs SchB can be quickly dispersed into the cytoplasm and take effect.

### **S**erum stability of the SchB/AP NPs

The stability of NPs in blood circulation is the primary condition for the passive targeting of NPs to tumor sites through EPR effects. Drugs are expected to have more excellent stability in the blood to maintain a longer duration of action in the body. To investigate the stability of the SchB/AP NPs, the NPs were placed in the medium containing serum for a total of 48 h. In Fig. 2d, the size of the NPs changed little within 48 h. This was indicated that SchB/AP NPs had weak interaction with serum proteins and showed a stable state.

The strong stability of SchB/AP NPs in serum may be because the NPs were not adsorbed by the proteins in serum and maintained a stable nanostructure. The stability of the nanoparticles ensures that the injected nanoparticles do not accumulate or precipitate in the blood circulation, thus maintaining their properties and drug activity. In addition, stable nanoparticles can achieve multiple functions: long circulation in the body, better-targeting ability, toxic side effects reduction, and therapeutic effect improvement [28].

### In vitro cytotoxicity

The inhibitory effects of the free SchB group and SchB/AP NPs on the proliferation of MS cells were shown in Table 2. The inhibitory effect of SchB/AP NPs (IC_50_: 10.54 ± 1.29 µg/mL) on MS cells was slightly smaller than that of free SchB (IC_50_: 7.31 ± 1.12 µg/mL).

This difference was explained by in vitro release results: the different cytotoxic effects of NPs and free groups were caused by the incomplete release of SchB in NPs within 48 h. Even though free SchB is highly toxic, free drugs are difficult to dissolve in water and lack an effective target site for accumulation in the body. In this case, the drug cannot effectively achieve the toxic effects of MS cells. It is worth noting that the IC_50_ (13.43 ± 0.65 µg/mL) of MCF-7 cells administered with AP NPs was significantly increased compared with the IC_50_ in MS cells previously reported by our research group. The drug resistance of MS cells is many times stronger than that of MCF-7 cells, and strong drug resistance is an important reason for the reduction of toxicity [29].

Li et al. used Vitamin E derivatives as drug resistance reversal agents to treat drug-resistant cancer cells together with chemotherapy drugs. They found that chemotherapy drugs were less toxic in resistant cancer cells than in normal cancer cells. Studies have found that the VE derivatives, while inhibiting the activity of drug-resistant cells, can reduce drug resistance and play a stronger inhibitory role [30]. Therefore, after loading SchB, NPs have great potential to reverse MDR and enhance the effect of ATRA on Pin1 inhibition.

**Table 2:** IC_50_ and RF of different ATRA preparations in MS cells

Groups	IC_50_ (µg/mL)	RF
Free SchB	7.31 ± 1.12	-
AP NPs	37.72 ± 0.55	-
SchB/AP NPs	10.54 ± 1.29^a^	3.68

^a^*p* < 0.05 vs. AP NPs

### Cellular uptake of the SchB/AP NPs

**Fig. 3:**
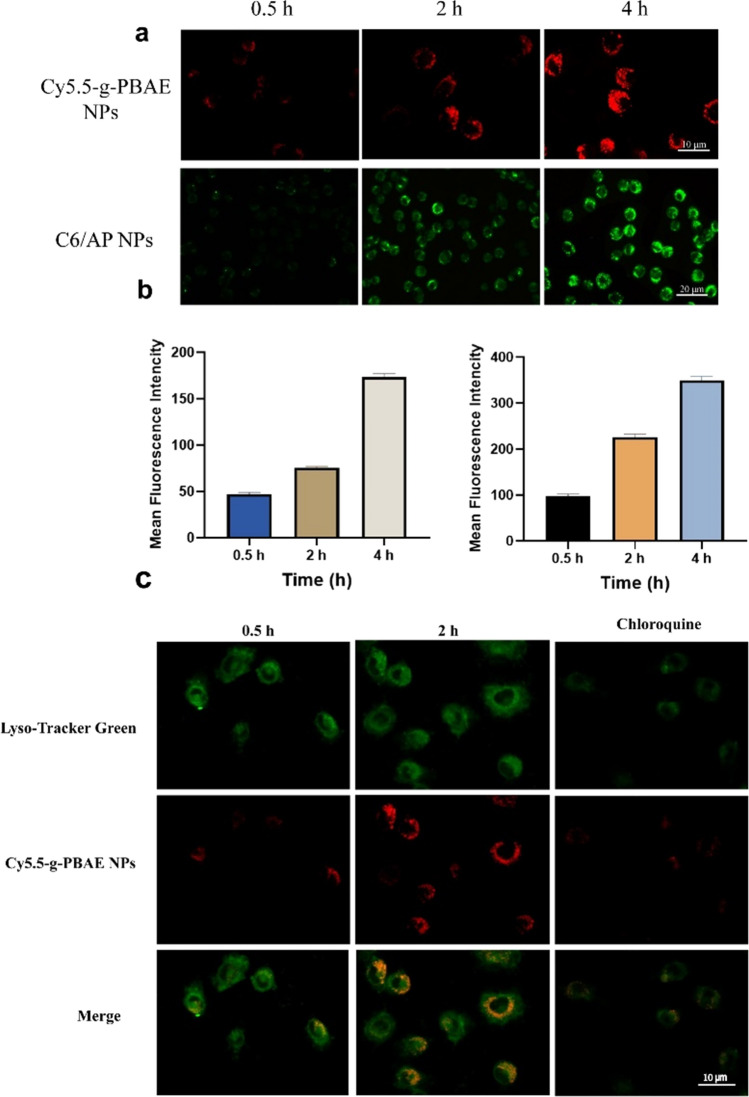
(**a**) Fluorescence microscope images of MS incubated with Cy5.5-g-PBAE NPs and C6/AP NPs (the scale bar was 10 and 20 μm); (**b**) Intracellular uptake intensity of Cy5.5-g-PBAE NPs. Intracellular uptake intensity of C6/AP NPs. (**c**) The images of Lysosome co-localization (the scale bar was 10 μm).

The uptake of Cy5.5-g-PBAE NPs and C6/AP NPs by MS cells was detected by fluorescence microscopy. In Fig. 3a and b, the fluorescence intensity was low within 0.5 h, and the amount of drug in the cell was minimal. When incubation time reached 2 h, enhanced red fluorescence intensity could be observed due to partial drug entry into cells. At 4 h, the fluorescence intensity in the cell was strengthened, and the amount of drug entering the cell increased. This proved that drug intake of MS cells was a time-dependent behavior with the extension of incubation time.

To demonstrate that the encapsulated drug can be transported into the cell through the carrier, the uptake of C6/AP NPs at different times was detected. C6 is similar to SchB in hydrophobic properties and can replace SchB as an encapsulated fluorescent drug. The green fluorescence intensity in MS cells also increased with the extension of incubation time.

Compared with free C6, nanoparticle-encapsulsated C6 showed excellent uptake performance. Li et al. incubated cells with free C6 and loaded C6 nanoparticles respectively, and found that weak fluorescence of free C6 could be observed only after 4 h, while the NPs group showed obvious green light within 1 h [31]. Nanocarriers can transfer hydrophobic molecules into cells more rapidly and release molecules into the cytoplasm with pH sensitivity. Compared with drug molecules, nano-loaded drug particles can increase the accumulation of local drug concentrations in tumor cells, increase bioavailability, and thus have higher therapeutic potential.

### Lysosome co-localizationIn

Fig. 3c, weak yellow fluorescence appeared in 0.5 h. This indicated that the color of Lyso-Tracker Green coincidated with Cy5.5. At 2 h, the red fluorescence increased significantly, and the yellow fluorescence was more obvious. However, the intracellular yellow fluorescence was significantly weaker in MS cells treated with chloroquine. Chloroquine is a drug that disrupts the acidic environment of the lysosome, thereby raising the pH of the lysosome. Therefore, chloroquine interfered with the release of acid-responsive drugs in lysozyme.

The results of the above uptake experiments have proved that blank NPs can carry SchB for cell uptake. The ingested NPs enter the early endosome and then the lysosome. Since PBAE is pH-sensitive, protonation of PBAE will cause lysosome rupture and further escape release [32].

### The mechanism of MS cells reversing MDR

#### Effect on the expression of drug-resistant protein P-gp

**Fig. 4:**
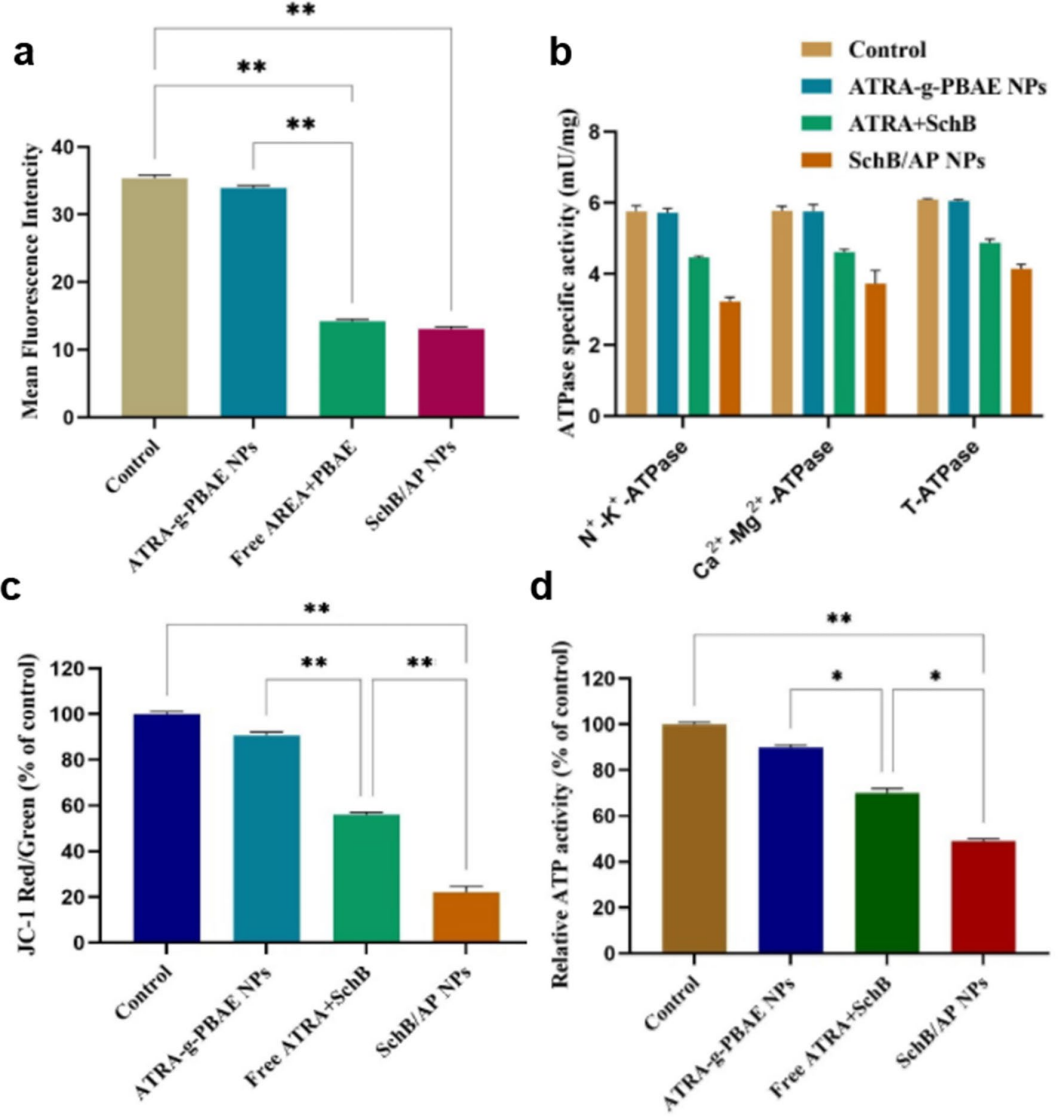
**a** The effect of the different formulations on P-gp expression in the MS cells; **b** The effect on ATPase, **c** effect of different groups on ΔΨm and **d** ATP content determination (*p < 0.05,**p < 0.01)

The results were shown in Fig. 4a, after MS cells were treated with ATRA-g-PBAE NPs group, there was no significant change in the produce of P-gp compared with the blank group. However, the expression of P-gp in MS cells treated with free ATRA + SchB decreased significantly (*p* < 0.01). Compared with the control group, the expression of P-gp was significantly reduced (*p* < 0.01) after treatment with SchB/AP NPs group. And the expression of P-gp, after free ATRA + SchB intervened, was similar to SchB/AP NPs group.

It has been reported that the chemical structure of SchB is similar to that of P-gp inhibitors and has been shown to inhibit P-gp expression [18]. Therefore, SchB/AP NPs has an approximate effect on reducing P-gp expression with free SchB.

P-gp is not only expressed in tumor tissues, but also widely distributed in other organs [33]. If the MDR reversing agent is combined with other P-gp, it will lead to the occurrence of drug side effects on the body. Hence, the use of pH-sensitive carrier encapsulation of SchB reversers is an effective way to improve drug targeting and reduce side effects.

#### Measurement of ATPase activity

The effects of SchB/AP NPs on Na^+^-K^+^-ATPase, Ca^2+^-Mg^2+^-ATPase and total ATPase (T-ATPase) activity were shown in Fig. 4b. Compared with control group and ATRA-g-PBAE NPs group, Na^+^-K^+^-ATPase, Ca^2+^-Mg^2+^-ATPase and T-ATPase activity in SchB and SchB/AP NPs groups were significantly decreased (*p* < 0.05). Mitochondria are crucial organelles and energy metabolism centers of cells, and their inner membrane is rich in enzymes related to energy metabolism, such as ATPase [34]. ATPase can hydrolyze ATP for the transport of P-gp to achieve energy supply [35, 36]. The ATPase activity of drug-loaded NPs was significantly lower than that of blank NPs. This suggests that SchB can reverse MDR by inhibiting ATPase activity and affecting P-gp energy supply and transport. However, the rate of passive diffusion of the free ATRA and SchB into the cells is different, and MS cells still maintain some resistance [37]. Therefore, the SchB/AP NPs group showed a stronger inhibitory effect.

Among them, Na^+^-K^+^-ATPase can promote ATP decomposition and maintain the balance of Na + and K + concentrations inside and outside cells, which can form resting potentials. Ca^2+^-Mg^2+^-ATPase can maintain intracellular energy stability by catalyzing ATP hydrolysis. When the activity of Na^+^-K^+^-ATPase and Ca^2+^-Mg^2+^-ATPase decreases, the internal and external flow of Na^+^ and Ca^2+^ of tumor cells decreases, resulting in abnormal transport function of tumor cells, and also interferes with mitochondrial uptake of Ca^2+^, which in turn causes abnormal energy metabolism in tumor cells [38]. Hence, the drug efflux function of MS cells could suppressed by SchB/AP NPs.

#### Assessment of mitochondrial membrane potential

The effects of AP NPs, free ATRA + SchB and SchB/AP NPs on ΔΨm are exhibited in Fig. 4c. Compared with the control group, ΔΨm decreased slightly after ATRA-g-PBAE NPs were incubated. Free ATRA and SchB, and SchB/AP NPs can significantly reduce ΔΨm in MS cells. Among them, the ΔΨm in the SchB/AP NPs group decreased significantly compared with the free drug group (*p* < 0.05).

JC-1 can determine the state of mitochondria and is a pivotal dye for detecting mitochondrial potential [39]. As the ΔΨm decreases, JC-1 was transferred from the mitochondrial matrix to the cytoplasm, and JC-1 changed from red fluorescence to green fluorescence [40]. A decrease in ΔΨm indicates that mitochondrial function has been disrupted and cannot release sufficient energy [41]. In the state of energy scarcity, various cells behaviors were blocked, especially P-gp mediated efflux. Furthermore, a decrease in ΔΨm indicated that cell death was about to occur. Free drugs captured by lysosomes are easily degraded under the action of their acidic environment and enzymes, resulting in reduced efficacy or even loss of efficacy [42]. It was due to the fact that after NPs co-deliver ATRA and SchB to MS cells, the intracellular concentration of ATRA was maintained by SchB and exerted cytotoxic effects.

#### Assay of ATP content

The change in intracellular ATP content after incubation in different groups was shown in Fig. 4d. Taking the control group as 100%, the ATP content of the other groups was calculated by the ratio respectively. MS cells experienced a slight decrease in ATP content after being given vector blank NPs. Compared with the free drug group, SchB/AP NPs caused a significant difference in ATP content reduction (*p* < 0.01). Moreover, the ATP content of cells from free drug was significantly lower than that from ATRA-g-PBAE NPs, indicating that the participation of SchB was a significant factor affecting the ATP content.

The transport of drugs requires the consumption of a large amount of ATP, so it causes a large amount of ATP production in cells. Especially for drug-resistant cells, P-gp-mediated cell excretion comes from rich ATP content [43]. The significant decrease in ATP may be due to SchB promoting the uptake process of NPs by cells, and intracellular ATP is consumed in large quantities. Moreover, SchB/AP NPs can inhibit cells activity, so the mitochondrial function is likewise inhibited [44]. Therefore, SchB/AP NPs have a significant ability to reverse the effect of MDR and have tremendous therapeutic potential for MS cells.

## Conclusion

In summary, we successfully constructed a novel dual-drug co-delivery SchB/AP NPs by grafting ATRA on PBAE and encapsulated MDR reversal agent SchB. We used CCD RSM to optimize the preparation of NPs, the SchB/AP NPs with good pharmaceutical properties were obtained. Because PBAE is a pH-sensitive carrier, the NPs has fast-release behavior and lysosomal escape ability at the same acidity as lysosome. Hopefully, we found that NPs was significantly cytotoxic and showed a reversal of cellular resistance to ATRA. In addition, we found that the effect of NPs reversing MDR may be accomplished by inhibiting P-gp and interfering with cellular energy metabolism. SchB/AP NPs, which reverse MDR based on co-delivery strategy to enhance the killing ability of cancer cells, provide a new strategy and research idea for anti-BCSCs resistance-related research.

Although the nano drug delivery system has shown excellent tumor targeted therapy effect. However, there are still limitations to our nanocarriers [45]. First, the loading efficiency of the carrier to the drug needs to be improved. Second, in vivo safety and biocompatibility have not been fully verified. Third, the mechanism of interaction between nanocarriers and tumor cells is still unclear and needs further study. We will explore these limitations in further research to overcome these obstacles. Besides, there are still many challenges in the clinical transformation of nanoparticles [46]. For example, the complexity of tumor biology and the scale of production quality control are important obstacles. In addition, the safety and biocompatibility of nanomaterials require more in-depth research and analysis, and long-term toxicity in vivo also needs to be studied.

## Data Availability

The datasets generated during and/or analysed during the current study are not publicly available due to some reasons but are available from the corresponding author on reasonable request.
